# Population admixture can enhance establishment success of the introduced biological control agent *Cryptolaemus montrouzieri*

**DOI:** 10.1186/s12862-018-1158-5

**Published:** 2018-03-27

**Authors:** Hao-Sen Li, Shang-Jun Zou, Patrick De Clercq, Hong Pang

**Affiliations:** 1 State Key Laboratory of Biocontrol, Ecology and Evolution, School of Life Sciences, Sun Yat-sen University, Guangzhou, 510275 Guangdong China; 2 Department of Crop Protection, Faculty of Bioscience Engineering, Ghent University, Ghent, Belgium

**Keywords:** Population admixture, Classical biological control, *Cryptolaemus montrouzieri*, Multiple introductions, Establishment success, Biological invasion

## Abstract

**Background:**

Introduced biological control agents have opportunities of population admixture through multiple introductions in the field. However, the importance of population admixture for their establishment success often remains unclear. Previous studies based on genetic markers have suggested a history of population admixture in the predatory ladybird *Cryptolaemus montrouzieri* Mulsant in China.

**Results:**

We tested whether population admixture may lead to fitness changes under laboratory conditions. We first found no mating barrier or strong bias between two parental populations, despite their differences in genetics and phenotypes. Then, our experimental evidence supported the hypothesis that admixed populations have a higher potential of establishment success, due to their superior reproductive ability, and hunger and cold tolerance inherited from one of the parental populations.

**Conclusions:**

We suggest that population admixture can be a breeding method to improve the performance of biological control agents, particularly when used in a classical biological control approach, but that consequences for potential invasiveness need to be considered.

## Background

In human-mediated introductions, exotic species are predicted to suffer from reductions in genetic diversity relative to their parental population during population founding events, reducing adaptive potential, and thus often leading to failed establishment in new areas [1]. One common way for aiding establishment success of an introduced species is to cross genetically differentiated populations (i.e. population admixture) [2]. Currently, an increasing number of studies document establishment of exotic species resulting from population admixture; these include plants [3], mussels [4], fishes [5], lizards [6], fruit flies [7] and ladybirds [8].

Population admixture is not necessarily beneficial across a broad spectrum of ecological contexts due to its different outcomes [2, 9]. On one hand, it can increase genetic diversity, create new genotypes and mask recessive deleterious mutations [10]. On the other, it may reduce mating success of parents (prezygotic isolation) and disrupt local adaptation [10]. Moreover, most studies dealing with population admixture have aimed at detecting multiple source populations from neutral genetic markers (e.g. microsatellite markers), but this method does not allow to ascertain whether population admixture occurred before or after the introduction, and whether population admixture benefits establishment [8]. In fact, some recent studies suggest that the importance of population admixture in determining establishment success has been overemphasized because non-admixed populations can perform as good as admixed populations [11, 12]. Other factors like propagule pressure can also increase fitness of introduced populations [13]. Therefore, it remains unclear how important population admixture is as a driver of establishment success.

Biological control involves the introduction (“classical biological control”) or augmentative release (“augmentation biological control”) of a natural enemy species for the control of a pest. When the natural enemy is not native to the area of release and multiple releases are done in the same or adjacent areas based on different populations in a classical biological control context, this may provide opportunities for population admixture. Based on genetic markers, population admixture events have been found in some field-released biological control agents, including the predatory ladybirds *Hippodamia convergens* [14] and *Harmonia axyridis* [8], and the parasitoid wasp *Trioxys pallidus* [15]. Further laboratory tests showed that population admixture enhanced fitness of certain biological control agents like *H. axyridis* [16, 17] and *Longitarsus jacobaeae* [18].

Here, we study another predatory ladybird, *Cryptolaemus montrouzieri* Mulsant (Coleoptera, Coccinellidae), which is native to Australia and has been introduced into at least 64 countries or regions for over 100 years for the control of mealybug pests [19]. In China, *C. montrouzieri* has been used in classical biological control programs for more than 60 years [20], but these programs seldom succeeded [21] and few populations could be found in wild. In previous studies on the genetic profile of populations used for biological control in China, we detected a strong genetic differentiation between the populations from the two main research institutes involved in this biological control program using microsatellite markers [22]. Based on Bayesian cluster analysis, we also predicted that a wild population collected from Guangdong, China, was probably derived from an admixture of these two laboratory populations [22], while no such admixture event was detected for other wild or lab-reared populations. From the above evidence, we proposed that population admixture of *C. montrouzieri* was caused by multiple introductions for biological control purposes, and we hypothesized that the admixed population of this ladybird had a higher fitness and potential for establishment than its parental populations. To test this hypothesis, we performed a population admixture experiment involving *C. montrouzieri* under laboratory conditions and evaluated the role of population admixture for fitness changes. We first assessed whether there was mating barrier that could prevent successful crosses between the two divergent Chinese populations mentioned above. Then, we estimated several life history traits of these two populations as well as of their admixed offspring. Lastly, we tested their performance under cold and hunger stress which they are expected to suffer upon release.

## Methods

### Insect culture

The two populations of *C. montrouzieri* used in the present study were maintained in the laboratories of Sun Yat-sen University (YY) and South China Agricultural University (CC) for over 10 years. Before the study, the populations were cultured under the same conditions for at least three generations. The ladybirds were fed on citrus mealybugs, *Planococcus citri* (Risso), which were reared on fruits of pumpkin, *Cucurbita moschata*. All insects and plants were kept in climate chambers at 25 ± 1 °C, a 50% relative humidity (RH), and a 14:10 (L:D) h photoperiod.

### Mating frequency

Experiments were conducted to determine whether females or males mated more or less with a genetically related versus distinct conspecific. Sexually mature and virgin females and males (> 5 days after emergence) were placed into two separate cages for 24 h. To distinguish between YY and CC individuals, the pronotum of each individual was marked using a marker pen. Then, one female and two males (YY♀ / YY♂ / CC♂ and CC♀ / YY♂ / CC♂) or one male and two females (YY♂ / YY♀ / CC♀ and CC♂ / YY♀ / CC♀) were placed in a Petri dish (diameter 10 cm, height 2 cm) with no food or water, and were given 3 h to allow multiple matings. Any mating event that took longer than 1 min was counted, and then the mating frequency was calculated. Circa 20 replicates were set up for each treatment. The percentages of replicates in which one or more mating events between YY and CC adults took place were calculated.

### Preparation of admixed populations

Adults of CC♀ / YY♂ and YY♀ / CC♂ (> 30 pairs for both) were placed in Petri dishes (diameter 10 cm, height 2 cm), and initiated the first generations of admixed populations CC♀ × YY♂ (CYF_1_) and YY♀ × CC♂ (YCF_1_). Adults of the two admixed F_1_-generations mated randomly within populations (CYF_1_♀ × CYF_1_♂ and YCF_1_♀ × YCF_1_♂) thus initiating several subsequent generations (YCF_n_ and CYF_n_).

To test the effect of population admixture on fitness changes, we compared the life history traits and hunger and cold tolerance of the admixed populations and their parental populations. We tested the performance of both admixed F_1_ and F_2_ populations, as population admixture often leads to a phenotypic breakdown in the F_2_ generation as a result of recombination disrupting co-adapted gene complexes or meiotic problems [23]. We also tested the admixed F_8_ populations for understanding the long-term consequences of population admixture.

### Life history traits

Several life history traits of the two parental (YY and CC) and six admixed (CYF_1_, YCF_1_, CYF_2_, YCF_2,_ CYF_8_ and YCF_8_) populations were investigated. About 60 third-instar larvae randomly collected within each population were placed individually in Petri dishes (diameter 4 cm, height 2 cm), and their development times from 4th instar to adult were measured. Newly emerged adults were sexed and weighed using an electronic balance (Sartorius BSA124S, Germany, ± 0.1 mg). Fecundity of ca. 30 newly emerged female / male pairs of each population was monitored. The number of deposited eggs of each pair was checked every 3 days for a total of 18 days. Larvae and adults of *C. montrouzieri* used in these experiments were provided with an ad libitum supply of citrus mealybugs.

### Hunger and cold tolerance

To estimate the lifespan of adults under conditions of starvation, about 30 adults (< 10 days after emergence) of each sex / population were randomly collected and placed individually (in order to prevent cannibalism) in a Petri dish (diameter 4 cm, height 2 cm) with no food or water. Their survival was checked every day and lethal times were calculated.

To estimate the survival rate of adults at low temperature, nine Petri dishes (diameter 10 cm, height 2 cm) containing ten adults (< 10 days after emergence) of each sex/population were placed in an incubator at 4 °C, which is the lowest average temperature in the winters of southern China. Throughout the cold exposure, the insects were kept in darkness and RH was not controlled. One Petri dish (of each sex/population) was taken from the incubator every day from the second to tenth day, and transferred back to 25 °C. Survival of the adults was determined after 24 h and lethal times were calculated.

### Statistics

Data on mating frequency, developmental time, number of deposited eggs, body weight and food consumption of females and males were first tested for normality and homogeneity of variances by a Kolmogorov-Smirnov test and Levene test, respectively. We used one-way analysis of variance (ANOVA) followed by Tukey tests to evaluate differences in body weight and food consumption of females and males. The developmental time and number of deposited eggs were tested using a Kruskal-Wallis test followed by a Steel-Dwass test due to the lack of normal distribution of the data. The significance level of all tests was set at *p* ≤ 0.05. The results from the lethal time experiments were analyzed using probit analysis in order to estimate the time required to kill 50% and 90% of the population (LTime_50_ and LTime_90_) at starvation or a temperature of 4 °C. Significant differences were identified by non-overlapping 95% fiducial limits. All analyses were performed using SPSS 21 (IBM SPSS Statistical, Chicago, USA) and R (R Development Core Team 2011).

## Results

### Mating frequency

**Fig. 1:**
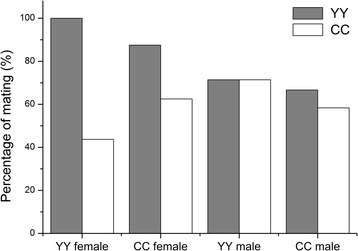
Percentages of replicates in which YY (grey) and CC (white) mated in the YY♀ / YY♂ / CC♂ (YY female), CC♀ / YY♂ / CC♂ (CC female), YY♂ / YY♀ / CC♀ (YY male) and CC♂ / YY♀ / CC♀ (CC male) treatments

The observed mating frequency of each replicate ranged from 0 to 11 matings over a 3-h period, with an average of 2.23. Mating percentages in the four treatment groups (Fig. 1) showed more self-breeding in YY♀ / YY♂ / CC♂ but more cross-breeding in CC♀ / YY♂ / CC♂. For the other two treatments, both YY and CC showed similar mating percentages ranging from 60% to 70%. Overall, our results suggest that there is no mating barrier or strong bias between the YY and CC populations.

### Fitness comparison between two parental populations

**Fig. 2:**
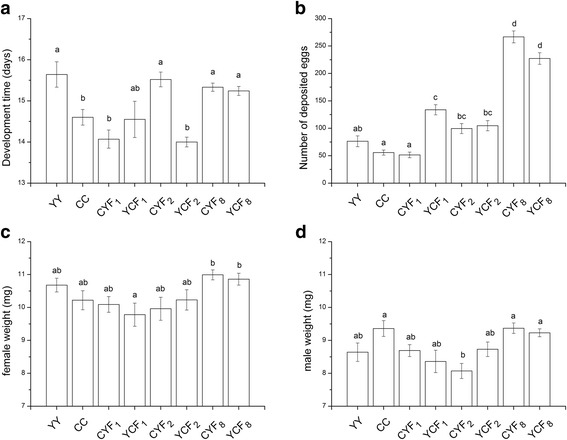
Life history traits of parental and admixed populations including **a** development time from 4th instar to adult, **b** number of deposited eggs in 18 days, fresh weight of newly emerged **c** female and **d** male adults, and. Bars (means ± SE) with the same letter are not significantly different (*p* > 0.05)

Four life history traits of the eight tested populations are reported in Fig. 2. Differences between the two parental populations were detected for development time and food consumption of females, with CC individuals developing faster (*p* = 0.0055, Steel-Dwass test) than those of YY. In addition, YY laid more eggs than CC but the difference was not statistically significant (*p* = 0.698, Steel-Dwass test).

**Fig. 3:**
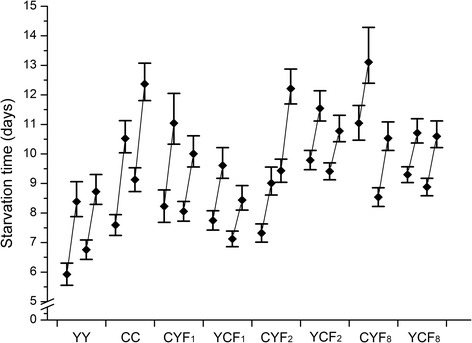
Lethal times for 50% and 90% mortality (LTime_50_ and LTime_90_) and their 95% fiducial limits of females and males from eight populations when exposed to starvation

**Fig. 4:**
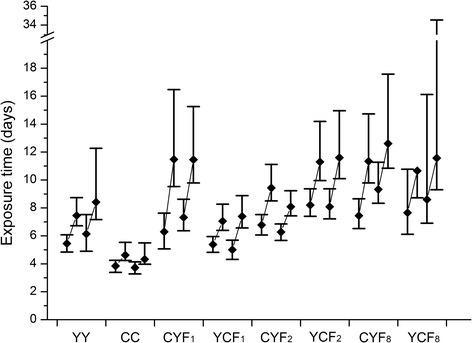
Lethal times for 50% and 90% mortality (LTime_50_ and LTime_90_) and their 95% fiducial limits of females and males from eight populations when exposed to a temperature of 4 °C

The LTime_50_ and LTime_90_ values under starvation and low temperature conditions are shown in Figs. 3 and 4, respectively. Here, we detected significantly different performances between the two parental populations. Starved CC adults of both sexes always lived longer than those of YY, whereas YY individuals performed better under cold stress than those of CC, based on non-overlapping fiducial limits of both LTime_50_ and LTime_90_.

### Fitness comparison between admixed and parental populations

The performances of admixed and parental populations were not always significantly different. For example, the development time of both population CYF_1_ and YCF_1_ was not significantly different from that of CC (*p* = 0.366 and 0.994, Steel-Dwass test). Also, CY and YC populations performed differently for certain parameters. For example, the CYF_2_ population was significantly different from YCF_2_ in terms of its development time (*p* < 0.001, Steel-Dwass test). Further, population admixture did not have significant effects on adult weight.

Among the tested traits, the population admixture had the most marked effect on the number of deposited eggs. From F_2_ to F_8_, CYF_2_ laid the lowest number of eggs among the admixed populations but this was still significantly higher than that in the CC population (*p* = 0.004, Steel-Dwass test). We found that admixed F_8_ populations laid significantly more eggs than and at least twice as much as the admixed F_1_, F_2_ and the parental populations (CYF_8_ vs YCF_1_: *p* < 0.001; YCF_8_ vs YCF_1_: *p* < 0.001, Steel-Dwass test).

For hunger tolerance, individuals of the admixed populations usually lived longer than those of the parental population YY, and longer than or as long as those of population CC, as indicated by the fiducial limits of both LTime_50_ and LTime_90_ values for females and males (except for YCF_1_ males and CYF_2_ females). For cold tolerance, female and male adults of all admixed populations lived significantly longer than those of the parental population CC, based on both LTime_50_ and LTime_90_ values.

## Discussion

### Laboratory evidence of population admixture

Our results meet several criteria for population admixture to play a role in establishment success [24]. First, we found that the two parental populations reared at two research institutes are significantly differentiated not only in terms of genotypes but also phenotypes. Second, there was no mating barrier, or even no apparent mating bias between the populations involved. The potential sperm competition with multiple mating was not tested in this study, but we consider that over their lifetime (ca. 3 months) *C. montrouzieri* adults of the two populations should have plenty of opportunity to generate admixed individuals under field conditions. Third, we found that in phenotypes characterized by development time, reproduction and stress tolerances, admixed populations can perform better than at least one of their parental populations. Below, we will discuss how the first and third criteria for population admixture may lead to establishment success in *C. montrouzieri*.

### Phenotypic differentiation among two genetically divergent populations

Differentiation among populations of biological control agents can be caused by drift (neutral process) and selection (non-neutral process) [25, 26]. Previously, we found that the populations YY and CC, as well as several other populations of *C. montrouzieri* from different parts of the world, were strongly differentiated genetically when using neutral genetic markers [22]. Also, we detected signals of episodic positive selection in their mitochondrial genomes [27]. Such rapid evolutionary process possibly leads to changes of several key traits that are under selection without reproductive isolation [28]. In this study, we detected that two genetically divergent but crossable populations, YY and CC, had certain significantly different phenotypes. Compared to YY, population CC had a shorter larval development time and longer survival time under starvation conditions but not at low temperature. The observed differences in phenotypic and genotypic traits of the different populations of *C. montrouzieri* used in biological control programs suggest that events of population admixture could possibly happen under multiple introductions.

### Heterosis and potential establishment success

The findings of our study indicate heterosis (vigor of population admixture) when simulating a probable event of population admixture of *C. montrouzieri* under laboratory conditions. In general, we found that admixed populations performed better than, or as well as, their parental populations for the tested traits. Moreover, with the exception of developmental time these superior traits of admixed populations were inherited from one of the parental populations (high reproductive ability and cold tolerance from YY, and hunger tolerance from CC) and could be long-term sustained.

The two parental populations involved in this study have throughout their laboratory rearing history been provided with ad libitum food and were continuously maintained under summer conditions (23 - 27 °C, long days). Therefore, they might have undergone less strong selective constraints resulting in the decline of hunger and cold tolerance. In contrast, the selective constraints these populations may have undergone after biological control releases are expected to be stronger. In a so-called “classical” biological control program, biological control agents are released with the objective of establishing populations for long-term control [29]. Establishment is thus a first and necessary step towards the success of such a biological control program. Cold tolerance is a critically important factor determining establishment success in cold or temperate areas. As *C. montrouzieri* originates from (sub)tropical regions [30], it is not expected to survive winters in colder climates [31]. Further, particularly when the natural enemy is more of a specialist feeder, like *C. montrouzieri,* it may not find sufficient prey or hosts after having been released in the field, hence the requirement for some level of starvation tolerance. Thus, the admixed populations in our study which performed better in terms of their reproduction and their ability to withstand hunger and cold stresses should have a higher potential of establishment success after biological control releases as compared with their parental populations. However, the data from our short-term laboratory studies do have limitations in predicting the long-term dynamics in field environments. Thus, further long-term experiments under field realistic conditions are recommended, considering the effects of amongst others multiple mating, prevailing climatic conditions and food availability, to improve our understanding of the effects of population admixture on the establishment potential of this and other biological control agents.

## Conclusions

In this study, we found that population admixture, which probably had also happened in field, increased fitness of an introduced biological control agent under laboratory conditions. Despite the limitations of the present laboratory study, our findings may have some implications for biological control applications. On the positive side, breeding techniques based on the intra-specific variation within a beneficial species are a new option for improvement of biological control agents [32]. Further, long-term laboratory-reared populations may be prone to loss of genetic diversity and inbreeding depression. Population admixture can increase genetic diversity and mask or purge recessive traits and may thus provide a fast avenue to breeding effective agents. On the other hand, there is growing concern about the environmental safety of biological control, particularly when non-indigenous natural enemies are being released [29]. Introductions of biological control agents with a high risk profile may lead them to become invasive [29, 33]. Such risk of increased invasiveness may be an unexpected outcome of population admixture [8]. To prevent potential invasiveness of non-indigenous biological control agents, invasive traits like wide, non-target food ranges [34] and strong dispersal ability [35] of admixed populations should be considered before release.

## References

[1] Bock DG, Caseys C, Cousens RD, Hahn MA, Heredia SM, Hübner S (2015). What we still don't know about invasion genetics. Mol Ecol.

[2] Rius M, Darling JA (2014). How important is intraspecific genetic admixture to the success of colonising populations?. Trends Ecol Evol.

[3] Keller SR, Taylor DR (2010). Genomic admixture increases fitness during a biological invasion. J Evol Biol.

[4] Gillis NK, Walters LJ, Fernandes FC, Hoffman EA (2009). Higher genetic diversity in introduced than in native populations of the mussel *Mytella charruana*: evidence of population admixture at introduction sites. Divers Distrib.

[5] Consuegra S, Phillips N, Gajardo G, de Leaniz CG (2011). Winning the invasion roulette: escapes from fish farms increase admixture and facilitate establishment of non-native rainbow trout. Evol Appl.

[6] Kolbe JJ, Glor RE, Rodríguez SL, Lara AC, Larson A, Losos JB (2004). Genetic variation increases during biological invasion by a Cuban lizard. Nature.

[7] Duchen P, Živković D, Hutter S, Stephan W, Laurent S (2013). Demographic inference reveals African and European admixture in the north American *Drosophila melanogaster* population. Genetics.

[8] Lombaert E, Guillemaud T, Thomas CE, Lawson Handley LJ, Li J, Wang S (2011). Inferring the origin of populations introduced from a genetically structured native range by approximate Bayesian computation: case study of the invasive ladybird *Harmonia axyridis*. Mol Ecol.

[9] Hahn MA, Rieseberg LH (2016). Genetic admixture and heterosis may enhance the invasiveness of common ragweed. Evol Appl.

[10] Verhoeven KJ, Macel M, Wolfe LM, Biere A (2011). Population admixture, biological invasions and the balance between local adaptation and inbreeding depression. Proc Biol Sci.

[11] Chapple DG, Miller KA, Kraus F, Thompson MB (2013). Divergent introduction histories among invasive populations of the delicate skink (*Lampropholis delicata*): has the importance of genetic admixture in the success of biological invasions been overemphasized?. Divers Distrib.

[12] Ordóñez V, Pascual M, Rius M, Turon X (2013). Mixed but not admixed: a spatial analysis of genetic variation of an invasive ascidian on natural and artificial substrates. Mar Biol.

[13] Simberloff D (2009). The role of propagule pressure in biological invasions. Annu Rev Ecol Evol Syst.

[14] Sethuraman A, Janzen FJ, Obrycki J (2015). Population genetics of the predatory lady beetle Hippodamia convergens. Biol Control.

[15] Andersen JC, Mills NJ (2016). Geographic origins and post-introduction hybridization between strains of Trioxys pallidus introduced to western North America for the biological control of walnut and filbert aphids. Biol Control.

[16] Facon B, Crespin L, Loiseau A, Lombaert E, Magro A, Estoup A (2011). Can things get worse when an invasive species hybridizes? The harlequin ladybird *Harmonia axyridis* in France as a case study. Evol Appl.

[17] Turgeon J, Tayeh A, Facon B, Lombaert E, De Clercq P, Berkvens N (2011). Experimental evidence for the phenotypic impact of admixture between wild and biocontrol Asian ladybird (*Harmonia axyridis*) involved in the European invasion. J Evol Biol.

[18] Szűcs M, Eigenbrode SD, Schwarzländer M, Schaffner U (2012). Hybrid vigor in the biological control agent, *Longitarsus jacobaeae*. Evol Appl.

[19] Kairo MKT, Paraiso O, Gautam RD, Peterkin DD. *Cryptolaemus montrouzieri* (Mulsant) (Coccinellidae: Scymninae): a review of biology, ecology, and use in biological control with particular reference to potential impact on non-target organisms. CAB Rev. 2013;8:005.

[20] Pu Z, He D, Deng D (1959). Studies on the breeding and utilization of *Cryptolaemus montrouzieri* and *Rodolia cardinalis*. J Sun Yat-sen Univ.

[21] Pang X, Li L (1979). Studies on the *Cryptolaemus montrouzieri* settling control of mealybug harm in Guangzhou and other place. Nat Enemies Insects.

[22] Li H-S, Liang X-Y, Zou S-J, Liu Y, De Clercq P, Ślipiński A (2017). New EST-SSR markers reveal strong genetic differentiation in native and introduced populations of the mealybug destroyer *Cryptolaemus montrouzieri*. Biol Control.

[23] Burke JM, Arnold ML (2001). Genetics and the fitness of hybrids. Annu Rev Genet.

[24] Wolfe LM, Blair AC, Penna BM (2007). Does intraspecific hybridization contribute to the evolution of invasiveness?: an experimental test. Biol Invasions.

[25] Hufbauer RA, Roderick GK (2005). Microevolution in biological control: mechanisms, patterns, and processes. Biol Control.

[26] Roderick GK, Navajas M (2003). Genes in new environments: genetics and evolution in biological control. Nat Rev Genet.

[27] Li H-S, Liang X-Y, Zou S-J, Liu Y, De Clercq P, Ślipiński A (2016). Episodic positive selection at mitochondrial genome in an introduced biological control agent. Mitochondrion.

[28] Whitney KD, Gabler CA (2008). Rapid evolution in introduced species, ‘invasive traits’ and recipient communities: challenges for predicting invasive potential. Divers Distrib.

[29] De Clercq P, Mason PG, Babendreier D (2011). Benefits and risks of exotic biological control agents. BioControl.

[30] Poorani J, Ślipiński A, Booth RG (2014). A review of the genus *Cryptolaemus* Mulsant (Coleoptera: Coccinellidae: Coccinellinae: Coccidulini) from new Guinea. Ann Zool.

[31] Maes S, Gregoire JC, De Clercq P (2015). Cold tolerance of the predatory ladybird *Cryptolaemus montrouzieri*. BioControl.

[32] Lommen ST, Jong PW, Pannebakker BA (2017). It is time to bridge the gap between exploring and exploiting: prospects for utilizing intraspecific genetic variation to optimize arthropods for augmentative pest control-a review. Entomol Exp Appl.

[33] Simberloff D, Stiling P (1996). Risks of species introduced for biological control. Biol Conserv.

[34] Louda SM, Pemberton RW, Johnson MT, Follett PA (2003). Nontarget effects-the Achilles’ heel of biological control? Retrospective analyses to reduce risk associated with biocontrol introductions. Annu Rev Entomol.

[35] van Lenteren JC, Babendreier D, Bigler F, Burgio G, Hokkanen HMT, Kuske S (2003). Environmental risk assessment of exotic natural enemies used in inundative biological control. BioControl.

